# TRPV1-mediated calcium signaling underlies the synergistic pro-apoptotic effects of lidocaine and melatonin in SH-SY5Y neuroblastoma cells

**DOI:** 10.3389/fonc.2026.1883694

**Published:** 2026-06-24

**Authors:** Ebru Aladag, Ishak Suat Ovey

**Affiliations:** 1 Department of Anesthesiology and Reanimation, Faculty of Medicine, Alanya Alaaddin Keykubat University, Alanya, Türkiye; 2 Department of Physiology, Faculty of Medicine, Alanya Alaaddin Keykubat University, Alanya, Türkiye

**Keywords:** apoptosis, calcium signaling, lidocaine, melatonin, mitochondrial dysfunction, neuroblastoma, oxidative stress, TRPV1 channel

## Abstract

Lidocaine, an amide-type local anesthetic, and melatonin, a multifunctional indoleamine with mitochondrial regulatory and anticancer properties, have each been reported to modulate cancer cell survival. However, whether these agents cooperatively promote apoptosis in neuroblastoma cells through transient receptor potential vanilloid 1 (TRPV1)-mediated calcium signaling remains insufficiently defined. This study investigated the individual and combined effects of lidocaine and melatonin on SH-SY5Y human neuroblastoma cells, focusing on TRPV1-dependent intracellular mechanisms. Intracellular Ca²^+^ responses were assessed using Fura-2-AM fluorescence, while apoptosis, reactive oxygen species (ROS) production, mitochondrial membrane potential (ΔΨm), and caspase-3/caspase-9 activities were evaluated using spectrofluorometric methods. The lidocaine + melatonin combination significantly increased cytosolic Ca²^+^ levels, ROS production, mitochondrial depolarization, caspase activation, and apoptosis compared with control and single-treatment groups. These responses were attenuated by capsazepine, supporting TRPV1-mediated Ca²^+^ influx as a central mechanism that appears to drive a Ca²^+^–mitochondria–ROS feed-forward axis leading to mitochondrial dysfunction and caspase-dependent apoptosis. These findings suggest that lidocaine and melatonin synergistically promote apoptosis in SH-SY5Y neuroblastoma cells through TRPV1-linked calcium-dependent pathways and provide a mechanistic basis for further investigation of anesthetic–adjunct interactions in translational oncology research.

## Introduction

Neuroblastoma (NB) represents the most common extracranial solid tumor in childhood, accounting for approximately 15% of all pediatric cancer-related deaths. Arising from neural crest-derived sympathetic nervous system progenitors, neuroblastoma exhibits remarkable clinical heterogeneity, ranging from spontaneous regression to aggressive metastatic disease resistant to conventional therapies (1). Despite advances in multimodal treatment strategies combining chemotherapy, surgical resection, and autologous stem-cell transplantation, the prognosis for high-risk neuroblastoma remains poor, with five-year survival rates below 50% and survivors often experiencing severe long-term sequelae from intensive treatment (1). These challenges underscore the urgent need for novel therapeutic approaches that can selectively induce cancer cell death while minimizing systemic toxicity.

Transient receptor potential vanilloid 1 (TRPV1), a non-selective cation channel permeable to Ca^2+^, has garnered increasing attention as a potential therapeutic target in cancer biology (2). Originally characterized as a nociceptor activated by capsaicin and noxious heat, TRPV1 is expressed in various cancer cell types, including neuroblastoma, where its activation triggers Ca^2+^ influx and downstream apoptotic signaling (3). Studies have demonstrated that TRPV1 activation in neuroblastoma cells leads to sustained [Ca^2+^]_i_ elevation, mitochondrial membrane depolarization, reactive oxygen species (ROS) accumulation, and caspase-dependent apoptosis (4). Furthermore, TRPV1-mediated Ca^2+^ signaling has been implicated in modulating cellular stress responses and apoptotic sensitivity in neural cells (4). These findings position TRPV1 as a promising molecular switch for selectively triggering cancer cell death through calcium-dependent mechanisms.

Lidocaine, a widely used local anesthetic of the amide class, has recently been recognized for its pleiotropic effects beyond sodium channel blockade (5). Evidence from *in vitro* cancer cell studies and experimental tumor models indicates that lidocaine can exert anti-proliferative and pro-apoptotic effects in several cancer cell types, including neuroblastoma-related models (6, 7). In cellular models, lidocaine has been reported to induce apoptosis through multiple mechanisms, including modulation of ion channel activity, disruption of mitochondrial function, oxidative stress, and activation of caspase pathways (6, 7). In the SH-SY5Y neuroblastoma cell line, lidocaine exposure has been associated with altered intracellular signaling and autophagy-related pathways (8). However, although lidocaine is widely used clinically as a local anesthetic and may also be administered systemically, extrapolation from *in vitro* findings to systemic clinical exposure requires caution because systemic lidocaine administration in humans requires careful monitoring due to potential central nervous system and cardiovascular toxicity. Therefore, the pharmacological background of lidocaine in the present manuscript is interpreted within the context of experimental cellular pharmacology rather than direct systemic clinical exposure.

Melatonin (N-acetyl-5-methoxytryptamine), an endogenous neurohormone primarily synthesized by the pineal gland, has demonstrated anticancer properties in preclinical studies across multiple malignancies (9). Beyond its well-established role in circadian rhythm regulation, *in vitro* and experimental studies suggest that melatonin can exert direct cytotoxic effects on cancer cells through diverse mechanisms, including induction of apoptosis, inhibition of proliferation, and modulation of oxidative stress (9, 10). In cancer cell models, melatonin has been shown to activate both intrinsic and extrinsic apoptotic pathways, upregulate pro-apoptotic proteins such as Bcl-2-associated X protein (Bax), downregulate anti-apoptotic B-cell lymphoma 2 (Bcl-2), and enhance caspase-3 and caspase-9 activities (11). Recent *in vitro* investigations have also revealed that melatonin can modulate transient receptor potential (TRP) channel activity, including TRPV1, thereby influencing Ca²^+^ homeostasis and apoptotic signaling (10). The ability of melatonin to penetrate cellular membranes readily and its generally favorable safety profile make it an attractive candidate for further investigation in combination strategies targeting neuroblastoma.

Despite the well-documented pro-apoptotic properties of lidocaine and melatonin in cancer and neuronal models, limited experimental evidence is currently available regarding their combined effects on neuroblastoma cells through TRPV1-mediated mechanisms (12). The rationale for investigating this dual-agent approach is based on several considerations. First, *in vitro* and experimental evidence indicates that both agents can modulate calcium-dependent apoptosis in cellular and neuronal models (13). Second, their distinct molecular mechanisms may converge on common downstream calcium-dependent signaling pathways, potentially resulting in synergistic activation of apoptotic cascades (3). Third, the established clinical use of lidocaine and the generally favorable safety profile of melatonin support further investigation of this combination; however, translation of the present *in vitro* findings requires careful consideration of clinically achievable concentrations, route of administration, and toxicity monitoring, particularly for systemic lidocaine exposure (5, 14). Furthermore, targeting TRPV1 as a central convergence point for dual-agent therapy may enhance selectivity toward cancer cells exhibiting elevated channel expression (12). Understanding the molecular basis of the cooperative pro-apoptotic effects of lidocaine and melatonin via TRPV1 may facilitate the development of novel therapeutic strategies for neuroblastoma and other neural crest-derived malignancies.

Accordingly, the present study was designed to investigate the pro-apoptotic effects of lidocaine and melatonin, administered individually and in combination, on SH-SY5Y neuroblastoma cells, with a specific focus on TRPV1-mediated signaling pathways.

## Materials and methods

### Reagents/stains

Trypsin–EDTA, fetal bovine serum (FBS), penicillin–streptomycin, Ham’s F12 medium, Dulbecco’s Modified Eagle Medium (DMEM), dimethyl sulfoxide (DMSO), and dihydrorhodamine-123 (DHR-123) were purchased from Sigma-Aldrich (St. Louis, MO, USA). Caspase-3 (Ac-DEVD-AMC) and caspase-9 (Ac-LEHD-AMC) fluorogenic substrates were obtained from Enzo Life Sciences (Lausanne, Switzerland). The APOPercentage™ apoptosis assay kit (including release buffer) was purchased from Biocolor Ltd. (Belfast, Northern Ireland). Fura-2 acetoxymethyl ester (Fura-2-AM) was purchased from Calbiochem (Darmstadt, Germany), and Pluronic^®^ F-127 was obtained from BioVision (San Francisco, CA, USA). Probenecid and the mitochondrial membrane potential dye 5,5′,6,6′-tetrachloro-1,1′,3,3′-tetraethylbenzimidazolylcarbocyanine iodide (JC-1) were purchased from Santa Cruz Biotechnology (Dallas, TX, USA).

### Cell culture

SH-SY5Y human neuroblastoma cells were obtained from the American Type Culture Collection (ATCC; Manassas, VA, USA). Cells were cultured in a 1:1 mixture of Ham’s F12 medium and DMEM supplemented with 10% FBS and 1% penicillin–streptomycin. Cultures were maintained in T25 culture flasks at 37 °C in a humidified incubator with 5% CO_2_, and the medium was replaced every 2–3 days. Cells at 75–85% confluence were used for experiments and treated as described below.

Following treatment, cells were washed with phosphate-buffered saline (PBS), detached using 0.25% trypsin–EDTA, collected into 15 mL conical tubes, and centrifuged at 100 × g for 5 min. After resuspension in fresh complete medium, cells were prepared for subsequent analyses.

### Study groups

SH-SY5Y cells were divided into seven experimental groups as follows:

Group 1 (Control): Cells were maintained in standard culture medium without exposure to lidocaine, melatonin, or capsazepine for 72 h.

Group 2 (Lidoc): Cells were treated with lidocaine (1 mM) for 24 h (8).

Group 3 (Lidoc + CPZ): Cells were treated with lidocaine (1 mM, 24 h) followed by capsazepine (CPZ; 0.1 mM, 30 min), a TRPV1 channel antagonist.

Group 4 (Lidoc + Mel): Cells were treated with lidocaine (1 mM) and melatonin (Mel; 0.2 mM) for 24 h.

Group 5 (Lidoc + Mel + CPZ): Cells were treated with lidocaine and melatonin for 24 h, followed by CPZ (0.1 mM, 30 min).

Group 6 (Mel): Cells were treated with melatonin (0.2 mM) for 24 h (15).

Group 7 (Mel + CPZ): Cells were treated with melatonin (0.2 mM, 24 h) followed by CPZ (0.1 mM, 30 min).

The lidocaine concentration of 1 mM was selected based on previous *in vitro* studies, including a study in SH-SY5Y cells, in which millimolar-range lidocaine concentrations have been applied to investigate intracellular signaling, autophagy, mitochondrial pathways, and apoptosis-related responses (8, 13). This concentration was used to obtain reproducible mechanistic responses in the SH-SY5Y cell culture model and should not be interpreted as a clinically achievable systemic plasma concentration.

For apoptosis, intracellular ROS, mitochondrial membrane potential, and caspase-3 and caspase-9 analyses, cells were additionally stimulated with capsaicin (Cap; 0.1 mM, 10 min) to activate TRPV1 channels prior to measurements. For calcium signaling experiments using Fura-2-AM, cells were stimulated with capsaicin at the 20th measurement cycle in the presence of 1.2 mM extracellular Ca^2+^ or Ca^2+^-free buffer.

### Measurement of intracellular free calcium concentration

Intracellular free Ca^2+^ concentration ([Ca^2+^]_i_) was measured using the UV-excitable calcium indicator Fura-2-AM, as previously described by Uğuz et al. and Martínez et al. (16, 17). Cells were loaded with Fura-2-AM (5 µM) in the presence of Pluronic^®^ F-127 (0.02%) at 37 °C for 45 min. After washing, fluorescence emission at 510 nm was recorded at alternating excitation wavelengths of 340 and 380 nm at 3 s intervals using a Synergy™ H1 microplate reader (BioTek, USA). Cap (0.1 mM) was applied to stimulate TRPV1 channels, and changes in [Ca^2+^]_i_ were expressed as the fluorescence ratio (F340/F380).

### Assessment of apoptosis and intracellular ROS production

Apoptotic cell death was evaluated using the APOPercentage™ assay (Biocolor Ltd.) according to the manufacturer’s instructions and previously published protocols (18, 19). This dye-uptake assay selectively stains apoptotic cells based on phosphatidylserine externalization. Following treatment, apoptotic SH-SY5Y cells were quantified spectrophotometrically at 550 nm using a Synergy™ H1 microplate reader (BioTek).

Intracellular ROS production was assessed using dihydrorhodamine-123 (DHR-123) as previously described (18, 19). Fluorescence intensities were measured at excitation/emission wavelengths of 488/543 nm using the same microplate reader.

### Caspase-3 and caspase-9 activity assays

Caspase-3 and caspase-9 activities were determined using the fluorogenic substrates Ac-DEVD-AMC and Ac-LEHD-AMC, respectively, according to previously published methods (18, 19). Following treatment, cells were lysed and incubated with the corresponding substrates. Fluorescence signals were measured at excitation and emission wavelengths of 360 and 460 nm, respectively, using a Synergy™ H1 microplate reader (BioTek). Enzyme activities were normalized to total protein content and expressed relative to control values.

### Mitochondrial membrane potential analysis

Mitochondrial membrane potential (ΔΨm) was assessed using JC-1 dye (1 µM) according to previously published protocols (18, 19). Following treatment, SH-SY5Y cells were incubated with JC-1 at 37 °C for 45 min. Green fluorescence was measured at excitation/emission wavelengths of 485/535 nm and red fluorescence at 540/590 nm using a Synergy™ H1 microplate reader (BioTek). Mitochondrial depolarization was expressed as the red/green fluorescence ratio and quantified relative to control values (18, 19).

### Statistical analysis

All data were expressed as mean ± standard deviation (SD). Statistical analyses were performed using GraphPad Prism version 7.04 for Windows (GraphPad Software, San Diego, CA, USA). Data distribution and variance homogeneity were assessed using GraphPad Prism prior to analysis. Comparisons among multiple groups were performed using one-way analysis of variance (ANOVA) followed by Tukey’s *post hoc* test for multiple pairwise comparisons. All experiments were conducted in at least three independent replicates, and each experimental group included a minimum of 10 samples unless otherwise stated. Differences were considered statistically significant at *P<*0.05.

## Results

### Effects of lidocaine and melatonin combinations on cytosolic calcium levels in SH-SY5Y cells

**Figure 1:**
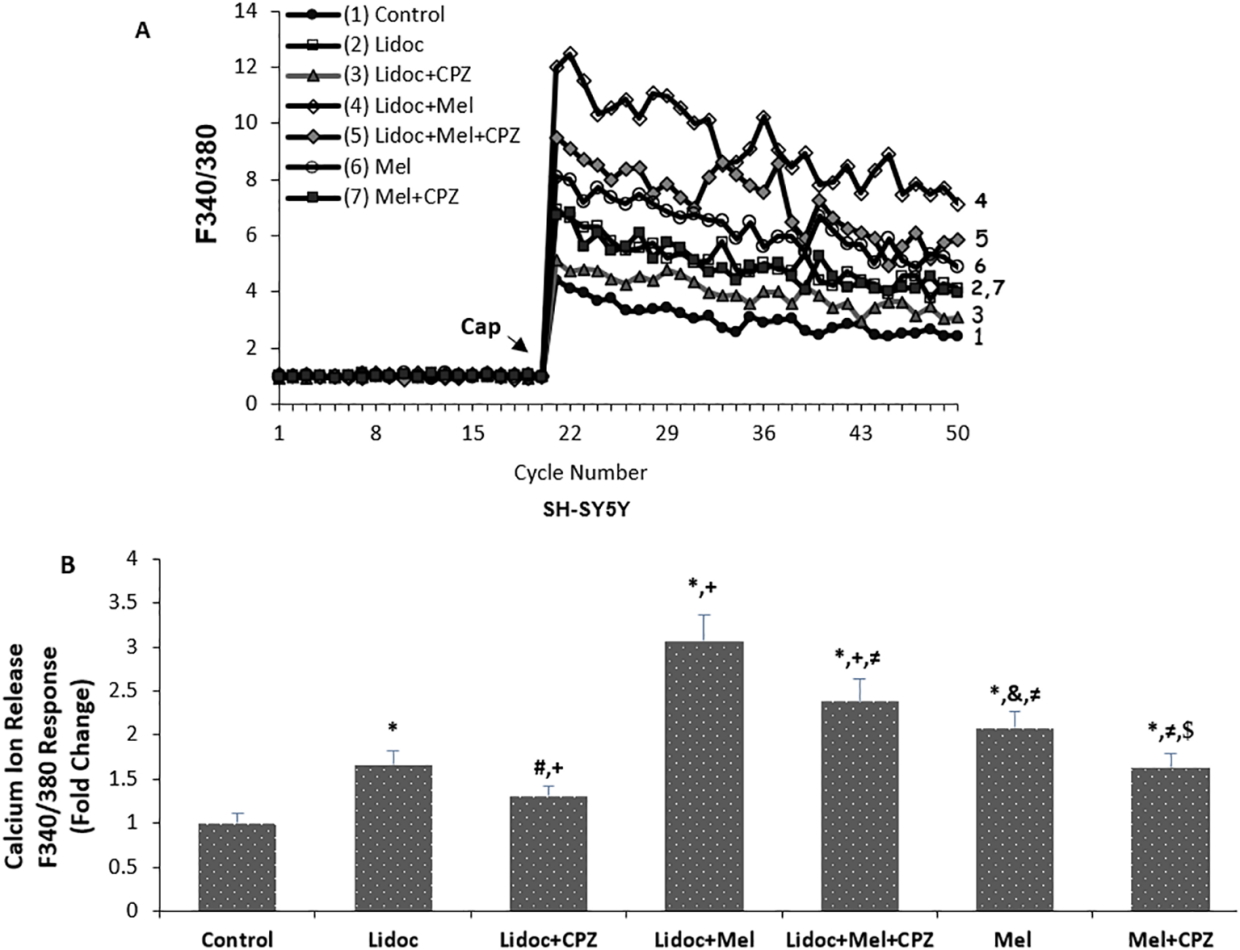
Effects of lidocaine and melatonin on TRPV1-mediated cytosolic Ca²^+^ levels in SH-SY5Y cells. **(A)** Representative traces of intracellular Ca²^+^ responses (F340/F380) following capsaicin (Cap, 0.1 mM) stimulation at the 20th cycle. **(B)** Quantitative analysis of Ca²^+^ responses (fold change) in different experimental groups. Cells were treated with lidocaine (Lidoc, 1 mM, 24 h) and melatonin (Mel, 0.2 mM, 24 h). Capsazepine (CPZ, 0.1 mM) was applied for 30 min prior to stimulation. Data are presented as mean ± SD from three independent biological replicates (n=3). *P<0.001 and ^#^P<0.05 vs. Control; ^+^P<0.001 and ^&^P<0.05 vs. Lidoc; ^≠^P<0.001 vs. Lidoc+Mel; ^$^P<0.05 vs. Mel group.

The effects of lidocaine and melatonin on intracellular cytosolic Ca^2+^ levels in SH-SY5Y cells are presented in **Figures 1A, B**. Cap, a TRPV1 channel agonist, and CPZ, a TRPV1 antagonist, were used to evaluate TRPV1-mediated Ca^2+^ influx.

As shown in **Figure 1B**, cytosolic Ca^2+^ concentrations were significantly increased in the lidocaine, melatonin, and lidocaine + melatonin groups compared with the control group (*P<*0.001). The lidocaine + melatonin group exhibited the highest Ca^2+^ response among all treatment groups (*P<*0.001 vs. lidocaine and melatonin). In addition, Ca^2+^ levels in the melatonin-treated group were significantly higher than those in the lidocaine-treated group.

Pre-treatment with capsazepine significantly reduced Ca^2+^ influx in the lidocaine + CPZ, melatonin + CPZ, and lidocaine + melatonin + CPZ groups compared with their respective non-CPZ groups (*P<*0.001), indicating attenuation of TRPV1-mediated calcium entry.

### Effects of lidocaine and melatonin combinations on apoptosis, intracellular ROS production, and mitochondrial depolarization in SH-SY5Y cells

**Figure 2:**
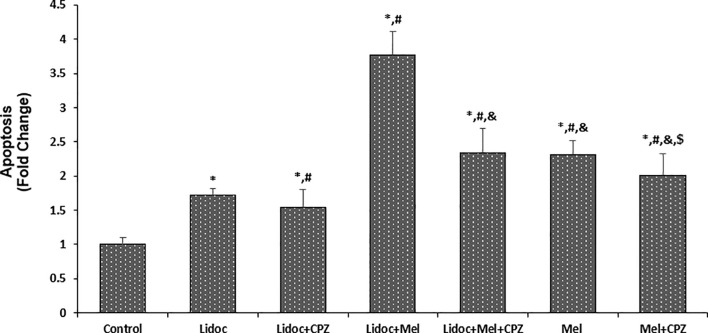
The effect of lidocaine (Lidoc, 1 mM, 24 h) and melatonin (Mel, 0.2 mM, 24 h) on apoptosis levels in SH-SY5Y cells. Cells were stimulated with capsaicin (Cap, 0.1 mM, 10 min) and inhibited by capsazepine (CPZ, 0.1 mM, 30 min) (mean ± SD, n=10). *P<0.001 vs. Control; ^#^P<0.001 vs. Lidoc; ^&^P<0.001 vs. Lidoc+Mel; ^$^P<0.001 vs. Mel.

**Figure 3:**
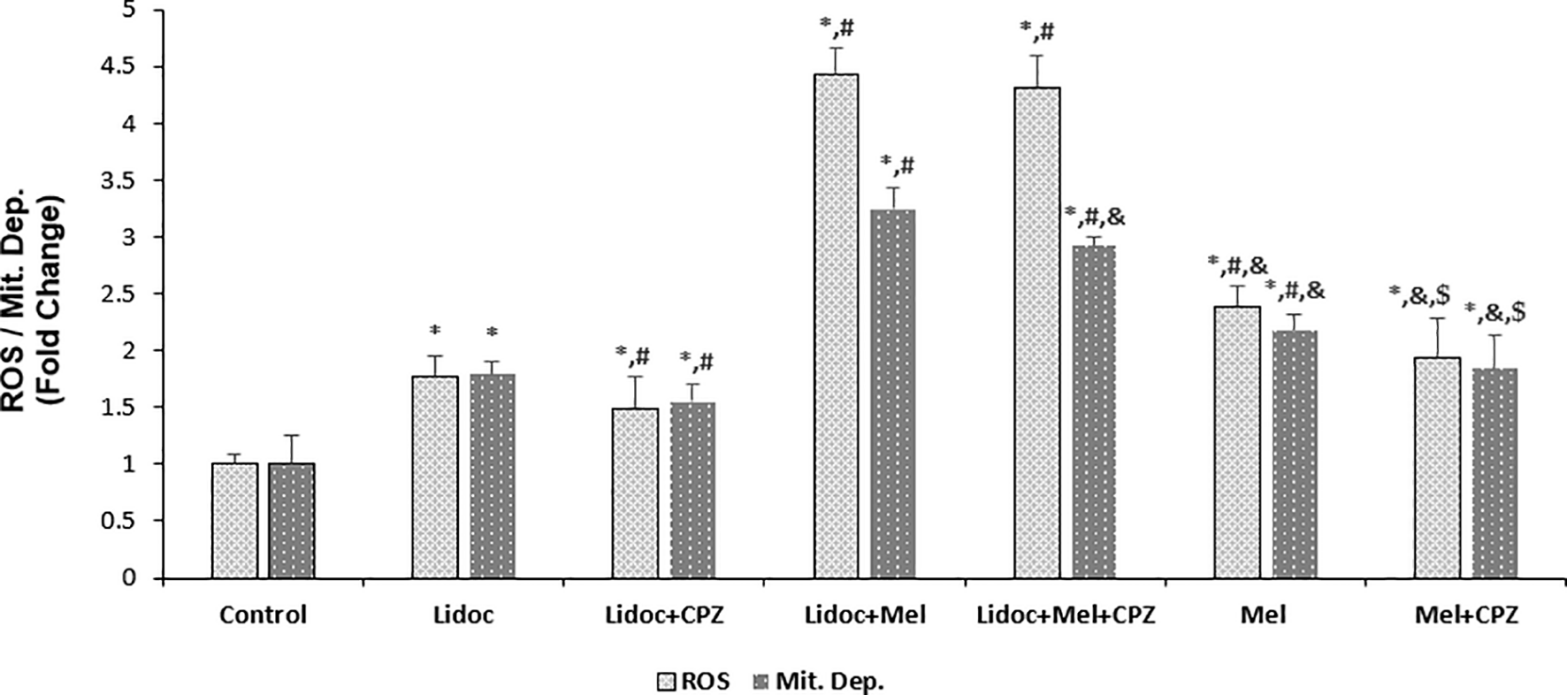
The effect of lidocaine (Lidoc, 1 mM, 24 h) and melatonin (Mel, 0.2 mM, 24 h) on ROS and mitochondrial depolarization levels in SH-SY5Y cells. Cells were stimulated with capsaicin (Cap, 0.1 mM, 10 min) and inhibited by capsazepine (CPZ, 0.1 mM, 30 min) (mean ± SD, n=10). *P<0.001 vs. Control; ^#^P<0.001 vs. Lidoc; ^&^P<0.001 vs. Lidoc+Mel; ^$^P<0.001 vs. Mel.

The effects of lidocaine and melatonin on apoptosis, intracellular ROS production, and mitochondrial depolarization in SH-SY5Y cells are presented in **Figures 2**, **3**. Apoptosis, ROS generation, and mitochondrial depolarization were significantly increased in the lidocaine, lidocaine + melatonin, and melatonin groups compared with the control group (*P<*0.001).

Among the treatment groups, the lidocaine + melatonin group exhibited the highest responses, showing significantly greater apoptosis, ROS, and mitochondrial depolarization than the lidocaine and melatonin groups (P<0.001). In addition, the melatonin group showed significantly higher levels than the lidocaine group for these parameters.

Pre-treatment with CPZ reduced apoptosis, ROS production, and mitochondrial depolarization in the corresponding groups (lidocaine + CPZ, melatonin + CPZ, and lidocaine + melatonin + CPZ) compared with their respective non-CPZ groups (P<0.001). However, in the ROS analysis, no statistically significant difference was observed between the lidocaine + melatonin and lidocaine + melatonin + CPZ groups.

### Effects of lidocaine and melatonin combinations on caspase-3 and caspase-9 activities in SH-SY5Y cells

**Figure 4:**
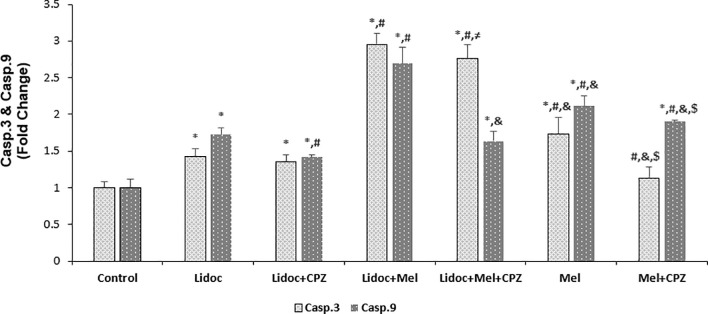
The effect of lidocaine (Lidoc, 1 mM, 24 h) and melatonin (Mel, 0.2 mM, 24 h) on caspase-3 and caspase-9 levels in SH-SY5Y cells. Cells were stimulated with capsaicin (Cap, 0.1 mM, 10 min) and inhibited by capsazepine (CPZ, 0.1 mM, 30 min) (mean ± SD, n=10). *P<0.001 vs. Control; ^#^P<0.001 vs. Lidoc; ^&^P<0.001 and ^≠^P<0.05 vs. Lidoc+Mel; ^$^P<0.001 vs. Mel.

The effects of lidocaine and melatonin on caspase-3 and caspase-9 activities in SH-SY5Y cells are presented in **Figure 4**. Caspase-3 and caspase-9 activities were significantly increased in the lidocaine, lidocaine + melatonin, and melatonin groups compared with the control group (*P<*0.001).

Among the treatment groups, the lidocaine + melatonin group exhibited the highest caspase-3 and caspase-9 activities, showing significantly greater levels than the lidocaine and melatonin groups (*P<*0.001). In addition, caspase activities in the melatonin-treated group were significantly higher than those in the lidocaine-treated group.

Pre-treatment with CPZ significantly reduced caspase-3 and caspase-9 activities in the lidocaine + CPZ, melatonin + CPZ, and lidocaine + melatonin + CPZ groups compared with their respective non-CPZ groups (*P<*0.001). However, in the caspase-3 analysis, no statistically significant difference was observed between the lidocaine and lidocaine + CPZ groups.

## Discussion

Local anesthetics and adjunctive agents have increasingly been recognized for their potential effects on tumor biology beyond their conventional roles in perioperative care (20). Lidocaine, in particular, has been shown to modulate intracellular signaling pathways and cellular survival mechanisms in neuroblastoma cells, including autophagy and apoptosis-related processes (8, 13). Similarly, melatonin, traditionally known for its chronobiotic and antioxidative properties, has emerged as a potent regulator of oxidative stress and mitochondrial function in cancer cells (21). Accumulating evidence suggests that melatonin can enhance chemotherapy-induced cytotoxicity and sensitize malignant cells to apoptotic stimuli (9, 19).

In this context, growing attention has been directed toward the involvement of TRPV1 channels in cancer cell apoptosis (2). TRPV1 channels, well established for their role in nociception and sensory signaling, also regulate intracellular calcium influx and mitochondrial homeostasis in malignant cells (2). Activation of TRPV1 induces calcium overload, mitochondrial depolarization, ROS production, and caspase cascade activation, ultimately leading to programmed cell death (22). These mechanisms highlight TRPV1 as a potential therapeutic target in oncology (2).

Within this mechanistic framework, our findings indicate that the combined administration of lidocaine and melatonin enhances apoptosis in SH-SY5Y neuroblastoma cells through TRPV1-mediated calcium signaling, mitochondrial dysfunction, and caspase activation, supporting previous observations that lidocaine modulates intracellular signaling and cell survival in neuroblastoma models (13). Furthermore, previous evidence suggests that lidocaine may exert antitumor effects by inhibiting cancer cell migration and invasion (20). These findings may offer mechanistic insights relevant to perioperative cancer management (23).

Importantly, the attenuation of lidocaine- and melatonin-induced calcium responses by capsazepine in the present study suggests that TRPV1 activation contributes to the observed intracellular Ca²^+^ influx. This interpretation is consistent with previous evidence demonstrating that lidocaine can activate TRPA1, a member of the TRP channel family, in nerve terminals, thereby supporting the ability of lidocaine to modulate TRP channel-dependent excitatory signaling (24).

In our SH-SY5Y neuroblastoma model, the increase in cytosolic Ca²^+^ following lidocaine or melatonin exposure, together with its significant reduction by capsazepine pre-treatment, supports the involvement of TRPV1-dependent calcium signaling rather than a purely nonspecific elevation of intracellular calcium (24, 25).

Furthermore, TRPV1 activation provides a mechanistic framework for understanding the synergistic pro-apoptotic interaction observed between lidocaine and melatonin. Lidocaine may act as an upstream activator or sensitizer of TRPV1-dependent calcium entry, while melatonin may complement and amplify this pathway by concurrently modulating mitochondrial susceptibility and intracellular oxidative stress. When administered together, these mechanisms appear to converge on a Ca²^+^–mitochondria–ROS axis: enhanced TRPV1-mediated Ca²^+^ influx promotes mitochondrial depolarization, ROS accumulation, and downstream caspase-9 and caspase-3 activation, while oxidative and mitochondrial stress may in turn further reinforce calcium-dependent apoptotic signaling in a feed-forward manner. This convergence provides a plausible mechanistic explanation for why the lidocaine + melatonin combination produced greater apoptosis-related responses than either agent alone, and why these effects were attenuated by capsazepine pre-treatment. Ultimately, such synergism may contribute to improved therapeutic responsiveness and reduced treatment resistance in neuroblastoma models.

From a perioperative medicine perspective, understanding how anesthetic and adjuvant agents influence cancer cell biology has important clinical implications. Increasing evidence indicates that perioperative interventions may affect long-term oncological outcomes (23). Lidocaine and other local anesthetics have demonstrated potential antimetastatic and antiproliferative properties in experimental and clinical studies (20). The adjunctive use of melatonin in perioperative settings may further enhance these effects, providing dual benefits in analgesia and tumor modulation (9).

In line with previous reports, our results suggest that the concomitant use of lidocaine and melatonin leads to increased intracellular ROS production and apoptosis compared with either agent alone, supporting the involvement of oxidative stress–mediated apoptotic mechanisms.

Despite these promising observations, several challenges remain for clinical translation. The concentrations used *in vitro* exceed plasma levels typically achieved under standard clinical conditions, highlighting the need for careful dose-optimization studies. Future investigations should focus on pharmacokinetics, safety profiles, and optimal administration schedules in perioperative and oncological settings. In addition, the long-term safety of melatonin administration in cancer patients must be considered (26).

## Limitations

This study has several limitations. First, the calcium signaling experiments were performed in three independent biological replicates. Although these experiments showed consistent response patterns across groups, including increased Ca²^+^ responses in the lidocaine + melatonin group and attenuation of these responses by capsazepine, additional independent replicates in future studies would further strengthen statistical confidence.

Second, a CPZ-only control group was not included in the present experimental design. Although CPZ was used as a pharmacological antagonist to assess the involvement of TRPV1 channels in the observed responses, the independent effects of CPZ on basal cytotoxicity or cellular signaling in SH-SY5Y cells were not evaluated in a separate experimental group. Future studies including a CPZ-only control group would further strengthen the interpretation of TRPV1-dependent effects.

Finally, the potential integration of lidocaine and melatonin with targeted therapies or immunotherapeutic agents warrants further investigation. Such multimodal strategies may enhance therapeutic efficacy while maintaining acceptable safety profiles in perioperative settings. Collectively, these findings provide a mechanistic foundation for future translational and clinical studies aimed at improving oncological outcomes.

**Figure 5:**
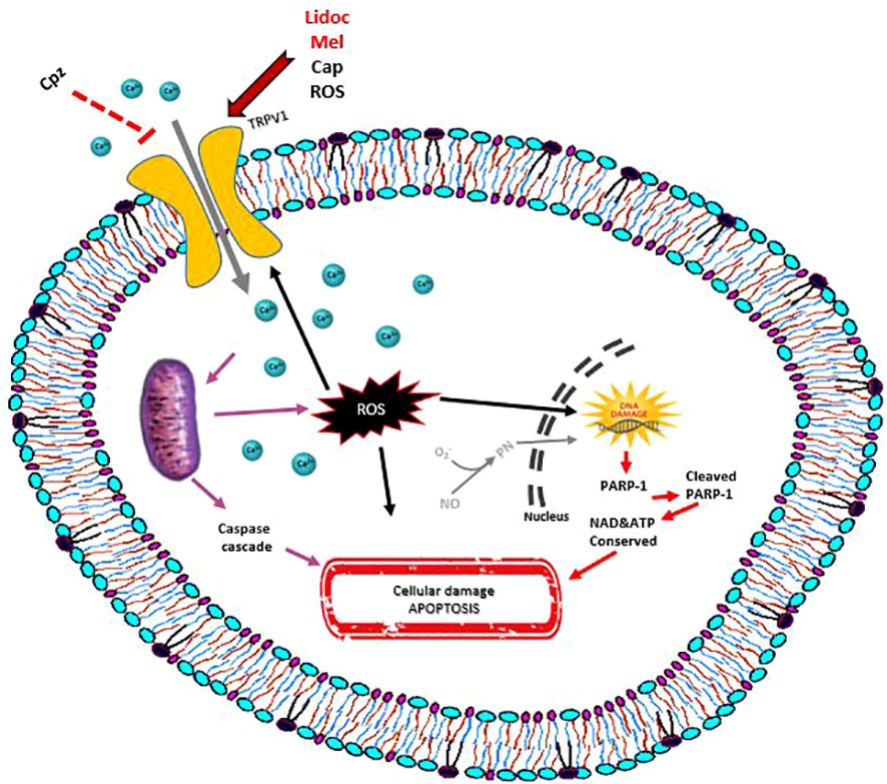
Graphical abstract summarizing the TRPV1 channel-dependent intracellular mechanisms by which lidocaine (Lidoc) and melatonin (Mel) synergistically promote apoptosis in SH-SY5Y neuroblastoma cells. Lidocaine, melatonin, and capsaicin (Cap) converge on TRPV1-linked signaling, resulting in TRPV1-mediated Ca²^+^ influx and increased cytosolic Ca²^+^ levels. Capsazepine (CPZ), a TRPV1 antagonist, inhibits this calcium entry. The elevated intracellular Ca²^+^ is taken up by the mitochondrion—the organelle depicted on the left side of the figure—triggering mitochondrial dysfunction and depolarization (ΔΨm↓). Both increased cytosolic Ca²^+^ and mitochondrial dysfunction promote reactive oxygen species (ROS) generation. ROS may further sensitize TRPV1 channels, establishing a feed-forward amplification loop that intensifies Ca²^+^ influx and downstream apoptotic signaling. Elevated Ca²^+^ and ROS together activate the caspase cascade, including caspase-3 and caspase-9, ultimately leading to cellular damage and apoptosis. In parallel, superoxide (O_2_•^-^) may react with nitric oxide (NO) to form peroxynitrite (ONOO^-^/PN), which contributes to nuclear DNA damage. DNA damage activates poly(ADP-ribose) polymerase-1 (PARP-1), and subsequent PARP-1 cleavage by activated caspases is associated with NAD^+^/ATP depletion, further promoting apoptotic cell death. The synergistic pro-apoptotic effect of the lidocaine + melatonin combination may arise from convergent activation of TRPV1-dependent Ca²^+^ influx, ROS accumulation, mitochondrial depolarization, and caspase activation, producing greater apoptotic signaling than either agent alone, consistent with the synergistic responses observed in the present study. Cap, capsaicin; CPZ, capsazepine; Lidoc, lidocaine; Mel, melatonin; ROS, reactive oxygen species; PARP-1, poly(ADP-ribose) polymerase-1; NAD^+^, nicotinamide adenine dinucleotide; ATP, adenosine triphosphate; PN/ONOO^-^, peroxynitrite; ΔΨm, mitochondrial membrane potential.

The proposed intracellular mechanism is summarized in **Figure 5**. Briefly, lidocaine and melatonin appear to converge on TRPV1-linked calcium signaling, leading to increased Ca²^+^ influx, mitochondrial dysfunction, ROS generation, and caspase-3/caspase-9 activation, as well as a potential ROS–TRPV1 feed-forward sensitization loop and downstream DNA damage/poly(ADP-ribose) polymerase-1 (PARP-1)-associated pathway, thereby providing a visual explanation for the synergistic pro-apoptotic effect observed in the lidocaine + melatonin combination group.

## Data Availability

The original contributions presented in the study are included in the article/supplementary material. Further inquiries can be directed to the corresponding author.
